# Supplementary material for: Gigahertz-rate thin-film lithium niobate receiver for time-bin quantum communication
Source: Light Sci Appl. 2026 May 18;15:237. doi: 10.1038/s41377-026-02306-5 (PMC13183875; doi:10.1038/s41377-026-02306-5)
Supplement: Supplementary file 1 — Supplementary information for: Gigahertz-rate thin-film lithium niobate receiver for time-bin quantum communication [file 41377_2026_2306_MOESM1_ESM.pdf]

Supplementary information for: Gigahertz-rate thin-film lithium niobate receiver for time-bin quantum communication

Andrea Bernardi[1,2,*], Marco Clementi[3], Marcello Bacchi[3], Matías Rubén Bolaños[4], Sara Congia[3,5], Francesco Garrisi[2], Andrea Martellosio[2], Marco Passoni[2], Alexander Wrobel[2], Costantino Agnesi[4,6], Giuseppe Vallone[4,6], Paolo Villoresi[4], Federico Andrea Sabattoli[2], Matteo Galli[3], and Daniele Bajoni[1]

[1]Dipartimento di Ingegneria Industriale e dell'Informazione, Università di Pavia, Via A. Ferrata 5, 27100 Pavia, Italy
[2]Advanced Fiber Resources Milan s.r.l., Via Fellini 4, 20097 San Donato Milanese, Italy
[3]Dipartimento di Fisica "A. Volta", Università di Pavia, Via A. Bassi 6, 27100 Pavia, Italy
[4]Dipartimento di Ingegneria dell'Informazione, Università degli Studi di Padova, Via Gradenigo 6B, 35131 Padua, Italy
[5]CEA LETI and University Grenoble Alpes, MINATEC Campus, F-38054 Grenoble Cedex, France
[6]Quantum Technologies Research Center, Università degli Studi di Padova, Via Gradenigo 6B, 35131 Padua, Italy
[*]Corresponding author. Email: andrea.bernardi01@universitadipavia.it

Supplementary Note 1: Secure key rate analysis

Both QKD with active and passive basis selection schemes implement the BBM92 protocol. In both cases, the two users perform measurements in two mutually unbiased bases, namely the X and Z bases for the passive case, and the X and Y for the active case. We perform the SKR computation for both the asymptotic regime and for finite block length for a more realistic scenario. We denote by $C_{|ab\rangle}$ the number of detected coincidence events between the two users when Alice and Bob project their measurements onto the states on the basis B with eigenstates $|a\rangle$ and $|b\rangle$, respectively, where $B : a, b \in \{Z : 0, 1; X : +, -; Y : R, L\}$. We denote p_B as the probability that Bob and Alice measure the states in the basis B. The measurement rates for the three mutually unbiased bases are:

$$
\begin{aligned}
R_X &= (C_{|++\rangle} + C_{|+-\rangle} + C_{|-+\rangle} + C_{|--\rangle})/\tau, \\
R_Y &= (C_{|RR\rangle} + C_{|RL\rangle} + C_{|LR\rangle} + C_{|LL\rangle})/\tau, \\
R_Z &= (C_{|00\rangle} + C_{|01\rangle} + C_{|10\rangle} + C_{|11\rangle})/\tau
\end{aligned}
$$

where τ is the time required to accumulate a block of key. The corresponding quantum bit error rates (QBER) are given by:

$$
\begin{aligned}
Q_Z &= \frac{C_{|01\rangle} + C_{|10\rangle}}{C_{|00\rangle} + C_{|11\rangle} + C_{|01\rangle} + C_{|10\rangle}}, \\
Q_X &= \frac{C_{|+-\rangle} + C_{|-+\rangle}}{C_{|++\rangle} + C_{|--\rangle} + C_{|+-\rangle} + C_{|-+\rangle}}, \\
Q_Y &= \frac{C_{|RL\rangle} + C_{|LR\rangle}}{C_{|RR\rangle} + C_{|LL\rangle} + C_{|RL\rangle} + C_{|LR\rangle}}.
\end{aligned}
$$

To obtain the SKR, in the passive basis choice experiment we take advantage of the intrinsic asymmetry of the receiver, with $p_Z > p_X$, to define the Z basis as the one used to generate secret key (so called *key basis*), and the X basis as the one used for parameter estimation (so called *check basis*). On the other hand, for the active basis choice experiment, since both measuring probabilities are equal, i.e. $p_X = p_Y = 0.5$, by construction, it is not straightforward to define one of the two basis as the key/check basis. Thus, we opted to divide the detected coincidences of each basis choice into two groups: 90% of the data in basis B to generate key in said basis, while the remaining 10% is used for parameter estimation on basis B', with $B \neq B'$. The

relative SKR is defined as follows for both passive and active basis choice configurations:

$$S_{pass} = S_Z \tag{S1}$$
$$S_{act} = S_X + S_Y \tag{S2}$$

where, in the asymptotic regime, each basis measurement contribution is given by[1]:

$$S_Z = R_Z[1 - H(Q_X) - fH(Q_Z)] \tag{S3}$$
$$S_Y = R_Y[1 - H(Q_X) - fH(Q_Y)] \tag{S4}$$
$$S_X = R_X[1 - H(Q_Y) - fH(Q_X)] \tag{S5}$$

where $H(p) = -p\log_2 p - (1-p)\log_2(1-p)$ is the binary Shannon entropy function used for the privacy amplification, and $f = 1.06$ accounts for error correction.

In the finite block size regime, we redefine the contribution of each basis to the SKR as[1,2]:

$$S_Z = R_Z[1 - H(Q'_{X,pass}) - fH(Q_Z)] - \log\frac{2}{\varepsilon_{cor}\varepsilon_{sec}^2} \tag{S6}$$
$$S_Y = R_Y[1 - H(Q'_{X,act}) - fH(Q_Y)] - \log\frac{2}{\varepsilon_{cor}\varepsilon_{sec}^2} \tag{S7}$$
$$S_X = R_X[1 - H(Q'_Y) - fH(Q_X)] - \log\frac{2}{\varepsilon_{cor}\varepsilon_{sec}^2} \tag{S8}$$

where ε_{cor}, ε_{sec} are the correctness and secrecy parameters respectively. Here, we assumed that $\varepsilon_{cor} = 10^{-10}$ and $\varepsilon_{sec} = 10^{-10}$. (it is important to note that the source-independent nature of EBQKD protocols renders the inefficiencies in the source irrelevant for the secret key analysis). The parameter Q'_B represents the adjusted QBER for basis B, accounting for the statistical fluctuations that arise due to the finite block size. In particular, $Q'_{X,pass}(Q'_{X,act})$ corresponds to the adjusted QBER when using X as the check basis for the passive (active) basis choice experiment. These upper bounds when using the Serfling inequality are given by:

$$Q'_{X,pass} = Q_X + \sqrt{\frac{(n_Z+1)\log(1/\varepsilon_{sec})}{2n_X(n_X+n_Z)}} \tag{S9}$$
$$Q'_{X,act} = Q_X + \sqrt{\frac{(n_Y+1)\log(1/\varepsilon_{sec})}{2n_X(n_X+n_Y)}} \tag{S10}$$
$$Q'_Y = Q_Y + \sqrt{\frac{(n_X+1)\log(1/\varepsilon_{sec})}{2n_Y(n_Y+n_X)}} \tag{S11}$$

where n_B accounts for the total coincidences in the basis B. Thus they are defined as:

$$n_X = C_{|++\rangle} + C_{|--\rangle} + C_{|+-\rangle} + C_{|-+\rangle},$$
$$n_Y = C_{|RR\rangle} + C_{|LL\rangle} + C_{|RL\rangle} + C_{|LR\rangle},$$
$$n_Z = C_{|00\rangle} + C_{|11\rangle} + C_{|01\rangle} + C_{|10\rangle}$$

Analogously, when using the Chernoff inequality, the adjusted QBER is defined as

$$Q'_{X,pass} = \frac{(n_Z+n_X)\Gamma^+_{n_X,\varepsilon_{PE}}(m_X/n_X) - m_X}{n_Z}, \tag{S12}$$
$$Q'_{X,act} = \frac{(n_Y+n_X)\Gamma^+_{n_X,\varepsilon_{PE}}(m_X/n_X) - m_X}{n_Y}, \tag{S13}$$
$$Q'_Y = \frac{(n_Y+n_X)\Gamma^+_{n_Y,\varepsilon_{PE}}(m_Y/n_Y) - m_Y}{n_X}, \tag{S14}$$

with ε_{PE} the failure probability of the parameter estimation step, and

$$\Gamma^+_{n,\varepsilon}(p) = \begin{cases} \gamma^+_{n,\varepsilon}(p) & \text{if } p \in \left[0, \frac{1-2\kappa_{n,\varepsilon}}{1+\kappa_{n,\varepsilon}}\right] \\ 1+\varepsilon & \text{otherwise,} \end{cases} \tag{S15}$$

where $\kappa_{n,\varepsilon} = \frac{2}{9n}\ln(1/\varepsilon)$ and $\gamma^{+}_{n,\varepsilon}(x)$ defined as

$$\gamma^{+}_{n,\varepsilon}(x) = \frac{1}{1+4\kappa_{n,\varepsilon}}\left[3\kappa_{n,\varepsilon} + (1-2\kappa_{n,\varepsilon})x + 3\sqrt{\kappa_{n,\varepsilon}\left(\kappa_{n,\varepsilon} + x - x^2\right)}\right].$$

Supplementary Note 2: Analysis optimization of detected events on Z basis

In the context of QKD with passive basis selection, the Z measurement bases discriminates whether photons are detected in the early or late time-bin. In our setup, the limited temporal resolution of the SNSPDs (characterized by a timing jitter of approximately 50 ps) leads to a non-negligible overlap between the detection distributions of the two time-bins, as shown in the statistical histogram of detection events in Fig. S1a.

To mitigate the resulting ambiguity in time-bin discrimination, we optimize the analysis of detection events by introducing a discarding window, centered between the early and late peaks with a tunable width. Detection events falling within this window are excluded, thereby reducing the QBER excluding ambiguous detections that could be incorrectly assigned to the wrong time-bin. However, this strategy also lowers the total number of valid detection events, as shown in Fig. S1b.

Fig. S1c illustrates how the SKR is affected by the trade off between reduced QBER and decreased detection rate. For the data presented in the Results section, we used an optimized discarding window width of 30 ps, which yielded the best SKR performance for both Chernoff and Serfling bounds.

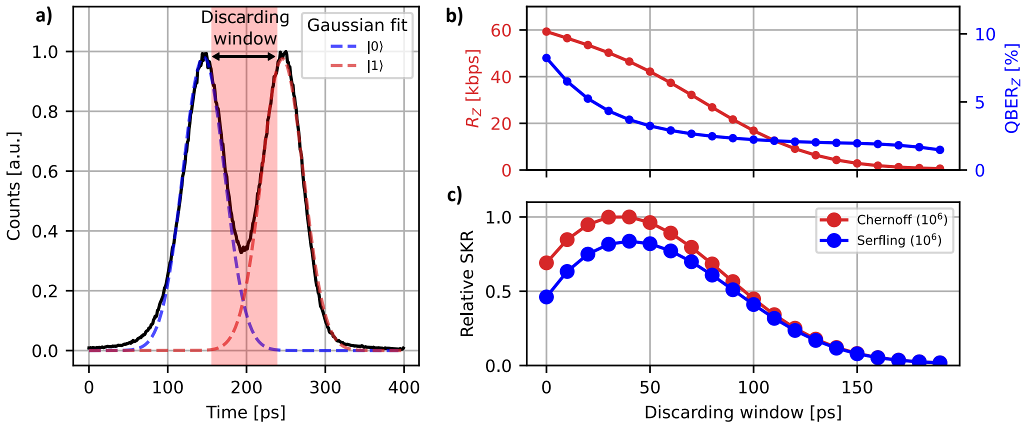

Fig. S 1 – Measurements on bases Z. a Histogram of time detection events for time-bin encoded photons in the Z basis for one QKD user. The detection time is relative to the laser pulse emission. **b** Coincidence rate and QBER between Alice and Bob of time-bin entangled photons in the Z basis, as a function of the discarding window width. **c** SKR as a function of the discarding window in the passive basis selection configuration. The optimal value is 30 ps for both bounds with a block size of 10^6.

Supplementary Note 3: QKD on variable fiber length

The QKD experimental implementation was also tested by varying the fiber channel length using spools of SMF28 optical fiber. The fiber spool was placed instead of the VOA, thus affecting only the Bob user's channel. This configuration was tested for the passive basis selection scheme. In standard single-mode fiber, the pulses which have a FWHM of 9.2 ps and a spectral bandwidth of 0.42 nm are subject to chromatic dispersion, which causes temporal broadening of the time-bin wavepacket as it propagates. In our case this effect will prevent to discriminate correctly in the basis Z the 100 ps separated time-bins, and in case of measurements in the interferometric bases X and Y to apply the correct switching inside the receiver device.

Fig. S2 shows the histogram detection events as a function of arrival time for both users in the Z basis. The temporal shape of Alice's detection events remains unchanged since its channel length is fixed and serves as a reference. In contrast, Bob's detection events broaden with increasing fiber length due to dispersion. This temporal broadening leads to greater overlap between the early and late time-bins, resulting in an increased QBER. To mitigate the effects of chromatic dispersion, and thus enable the practical deployment of the QKD system over long fiber links, a dispersion compensation strategy should be envisioned, for example by the use of dispersion compensating fiber or chirped fiber-Bragg gratings. We emphasize that these strategies, common to standard telecommunication systems, do not introduce, in principle, any disturbance to the quantum channel, except for a typically mild insertion loss.

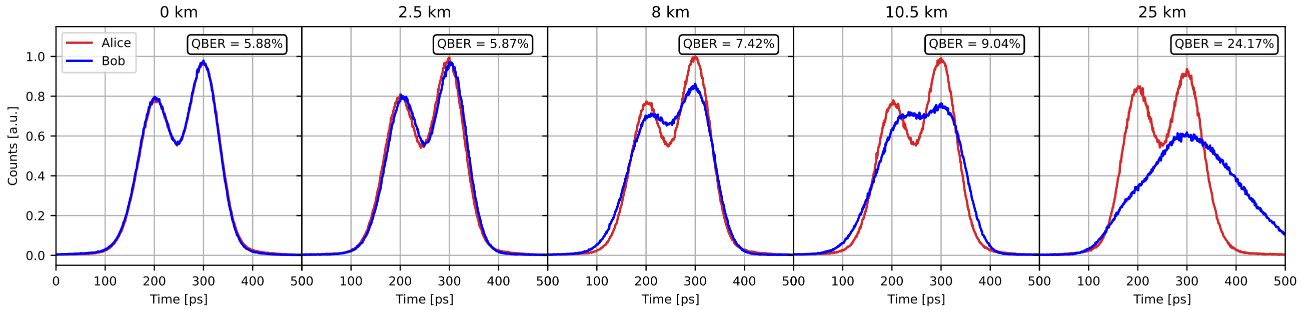

Fig. S 2 – Dispersion effect on variable fiber length. Normalized detection counts in the Z basis for the two QKD users, measured at different fiber-link lengths for Bob. The time reference on the x-axis is relative to the pump pulse emission. As the fiber length increases, the pulses broaden, reducing the temporal distinguishability of Bob's time-bin states.

Supplementary Note 4: Source characterization and pump power optimization

An integrated silicon waveguide fabricated by CEA Leti was used to generate entangled photon pairs. Measuring 16 mm in length, it featured a rib geometry and access via grating couplers. The total insertion loss, including input/output coupling and propagation loss at the pump wavelength of 1550.92 nm, is 5.5 dB. Fig. S3a shows the characterization of the entangled photon generation by means of SFWM, with signal and idler photons at ITU channels 28 and 38, respectively, as a function of the pump pulse energy. The pulse energy inside the waveguide is estimated from the input pump power, taking into account equal input/output coupling losses and the pulse repetition rate.

The retrieved generation rate in waveguide shows the expected quadratic dependence on the pulse energy. The measurement range is limited by the maximum allowed average coupled pump power (< 15 dBm), which prevented reaching the saturation regime. The Coincidence to accidental (CAR) ratio was computed as $CAR = (C_C - C_A)/C_A$ where C_C and C_A are relatively the detection events associated to the entangled photons, and the accidental detection events associated to coincidences delayed by multiples repetition rate. The integration window conditions were equals for both the detection contributes.

For QKD applications, the pump power should be selected to maximize the final SKR[3]. While a higher mean photon number increases the coincidence rate, it also leads to an increase in the QBER. As shown in Fig. S3b, the SKR for the active basis selection QKD scheme is plotted as a function of the internal source generation probability. The maximum SKR is achieved at an internal pair generation probability of approximately 0.7%. The results reported for the active-basis selection configuration are obtained using a mean photon number of approximately 0.2%, leading to the possibility of further SKR improvements by optimizing the generation rate.

In the case of the passive QKD scheme, a similar optimization can be applied. However, in our experiment we were limited by the maximum detection rate of the SNSPDs (~ 1.5 MHz) for measurements in the Z basis.

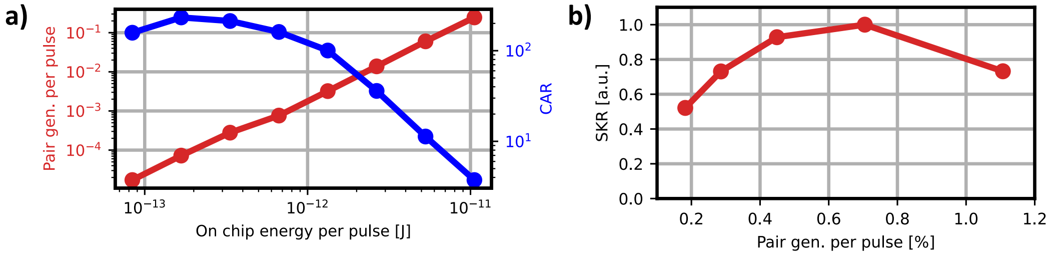

Fig. S 3 – Source characterization. **a** Internal waveguide pair-generation probability in ITU channels 28 and 38 (red), and coincidence-to-accidental ratio (CAR) (blue), as a function of the pump pulse energy. **b** Normalized SKR of the QKD protocol with active basis selection as a function of the internal waveguide pair-generation probability per pulse.

Supplementary Note 5: Additional characterization of the integrated photonic platform

We present additional test measurements of the TFLN technology used in this work to provide further insight into the performance of the integrated optical circuit. In particular, we report statistical measurements of components fabricated on the

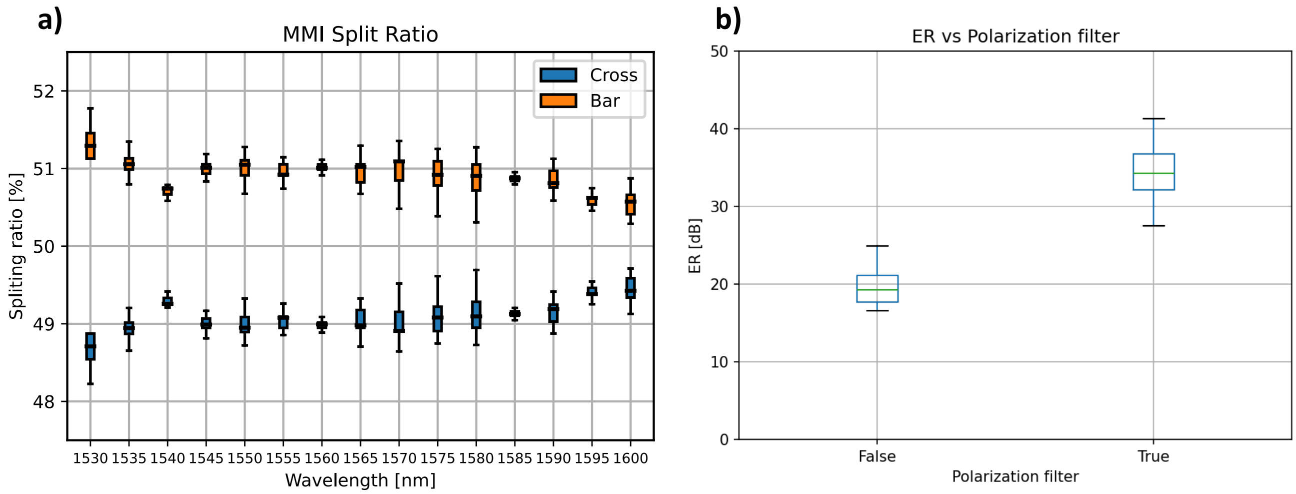

Fig. S 4 – TFLN components characterization. **a** Statistical characterization of the power splitting balance of standalone 2×2 MMIs fabricated on the same wafer as the presented device and featuring identical geometry. **b** Statistical distribution of the measured extinction ratio (ER) for interferometric structures fabricated on the same wafer and featuring the same optical components as the tested device, realized with and without an integrated polarization-splitter rotator (PSR). In both panels box rectangles extend from the 25th to 75th percentile, middle line shows the median and external lines show the farthest data point lying within 1.5x the inter-quartile range from the box.

same wafer as the device under test. These include standalone 2×2 MMI components and interferometric devices with an optical design similar to that of the realized device. Two sets of interferometric devices were fabricated to allow for a performance comparison based on the presence or absence of an on-chip polarization-splitter rotator (PSR) utilized as a polarization filter. These test measurements enable the identification and exclusion of factors that may limit interferometric visibility, which in turn affects the achievable quantum interference and the resulting QBER in the two interferometric bases X and Y.

The results obtained from the tested MMI components are shown in Fig. S4a. In the tested wavelength range from 1530 nm to 1600 nm, the MMIs exhibit a flat power imbalance of approximately 1%, corresponding to a theoretical extinction ratio (ER) of at least 34 dB. This level of imbalance alone, therefore, does not represent the dominant limitation to the visibility observed in our experiments.

Fig. S4b reports the ER obtained from interferometric structures featuring the same optical components as the present device, realized in two configurations: with and without an integrated PSR acting as a polarization filter to suppress the unwanted TM mode. The data reveal a clear improvement in the achieved ER of more than 10 dB when the PSR is present, providing evidence that polarization-mode mixing is a primary contributor to visibility degradation.

In conclusion, the main factors limiting the quantum interferometric visibility in the present device are the unbalanced losses intrinsic to the second-stage interferometer and polarization-mode mixing. In our implementation, the differential propagation loss is estimated to be approximately $1.3\,\mathrm{cm} \times 0.2\,\mathrm{dB\,cm^{-1}}$ in the longer arm and about 2% (~ 0.1 dB) in the shorter arm, leading to a small amplitude asymmetry between the interferometer paths. Residual TE–TM coupling further contributes to visibility degradation, as evidenced by the improved ER observed in devices incorporating an on-chip polarization filter.

References

1. Yin, J. *et al.* Entanglement-based secure quantum cryptography over 1,120 kilometres. *Nature* **582**, 501–505 (2020).

2. Tomamichel, M., Lim, C. C. W., Gisin, N. & Renner, R. Tight finite-key analysis for quantum cryptography. *Nature communications* **3**, 634 (2012).

3. Ma, X., Fung, C.-H. F. & Lo, H.-K. Quantum key distribution with entangled photon sources. *Physical Review A—Atomic, Molecular, and Optical Physics* **76**, 012307 (2007).
